# Predicting progressive vision loss in glaucoma patients using functional principal component analysis and electronic health records

**DOI:** 10.3389/fopht.2025.1632827

**Published:** 2025-11-19

**Authors:** Rithvik Krishna Donnipadu, Maxim Sivolella, Cody Carroll, Sophia Y. Wang

**Affiliations:** 1 Data Institute, University of San Francisco, San Francisco, CA, United States; 2 Department of Mathematics and Statistics, University of San Francisco, San Francisco, CA, United States; 3 Department of Ophthalmology, Byers Eye Institute, Stanford University, Palo Alto, CA, United States

**Keywords:** visual field, electronic health record, glaucoma, machine learning, longitudinal data analysis, functional principal component analysis

## Abstract

**Background:**

Glaucoma is a leading cause of irreversible blindness worldwide. Predicting a patient’s future clinical trajectory would help physicians personalize management. We present a novel approach for predicting patient visual field (VF) progression by combining Functional Principal Component Analysis (FPCA) with electronic health record (EHR) data.

**Methods:**

We identified glaucoma patients using diagnosis codes who had >=3 VF tests. We developed a 2-stage modeling pipeline: 1) Patients were split 80:10:10 into train, validation, and test sets and classified as fast-progressors or slow-progressors. 2) FPCA was used to predict mean deviation (MD) trajectories over 10 years after the baseline year of VF exams, using the first 2 principal components. To make predictions, the model uses up to one year of baseline VF and EHR data as input, but can flexibly make predictions from as few as a single VF test.

**Results:**

15,764 VF tests belonging to 2,372 patients were included, of which 8.92% of eyes were fast progressors. On the held-out test set, predictions over 10 years of future MD trajectories using baseline VF and EHR clinical data yielded an R^2^ of 0.646 and an RMSE of 3.67 for fast-progressors, and an R^2^ of 0.728 and an RMSE of 3.09 for slow-progressors. Performance was higher when predicting over the near term (fast progressors: year 1 R^2^ 0.920, RMSE 1.83; year 5 R^2^ 0.515, RMSE 4.26; slow progressors: year 1 R^2^ 0.918, RMSE 1.771; year 5 R^2^ 0.717, RMSE 3.12).

**Conclusion:**

A novel modeling approach combining FPCA with clinical data from EHR was able to model longitudinal clinical trajectories of glaucoma patients. This method is well-suited for modeling longitudinal healthcare data, handling sparse and irregular observation schedules with a varying number of inputs.

## 1 Introduction

Glaucoma is a chronic, progressive disease of the optic nerve and a leading cause of irreversible blindness worldwide (1). As glaucoma progresses, “blind spots” which are commonly asymptomatic in early stages can become large and debilitating. Treatment for glaucoma is aimed at slowing disease progression so patients maintain quality of life. Glaucoma is monitored through visual field (VF) testing which detects abnormal blind spots by presenting light stimuli of varying intensities in different locations while recording patient responses. Results are summarized using a global metric called mean deviation (MD), a measure of light sensitivity in decibels (dB) averaged over tested locations and compared to age-specific norms. Lower MD values indicate vision loss. While some patients remain stable for long periods, a small proportion are fast progressors who lose more than 1 dB/year of MD (2–5). Since VF tests are typically performed annually (6) and results can be noisy (7, 8), detecting progression is a difficult task requiring years of follow-up. If physicians could accurately predict a patient’s future VF trajectory, they could create more personalized treatment plans, particularly for slowing accelerated glaucoma progression.

Previous work has sought to predict VF mean deviation at specific future timepoints (9, 10) or classify patients as fast or slow progressors using initial test results (11, 12). While some efforts have yielded models with strong predictive power, limitations remain in their application to a clinical setting (13). Many previous approaches rely on a substantial number of baseline visual fields, requiring years of testing to make predictions (10, 14–16). Additionally, many architectures are inflexible with regard to the required number or temporal spacing of VF tests (9–11, 15, 17), which means predictions cannot be updated easily as new data is gathered. Furthermore, most architecture designs predict outcomes at specific timepoints (e.g., 1, 2, 3, etc. years in the future) (16). These predictions require different models for each timepoint. Finally, it has been shown that clinical data can help inform VF progression prediction (15, 18). However, many models lack integration of clinical data now widely available via electronic health records (EHR) (19, 20).

The purpose of this study was to predict glaucoma patients’ future VF trajectories using a novel approach that combines EHR clinical data inputs with Functional Principal Component Analysis (FPCA). FPCA is a longitudinal technique that estimates a population-level mean trajectory and its major variations, leveraging shared information across subjects to predict individuals’ future trajectories. Our approach constructs a continuous curve to predict a patient’s MD value over 10 years, requiring only one baseline VF. We further integrate EHR data, including demographics, exam results, diagnoses, and medications, to enhance prediction accuracy. Combining FPCA with clinical data represents an innovative approach to modeling longitudinal outcomes and can potentially improve management of glaucoma and other chronic progressive diseases.

## 2 Methods

### 2.1 Data source and study population

**Figure 1:**
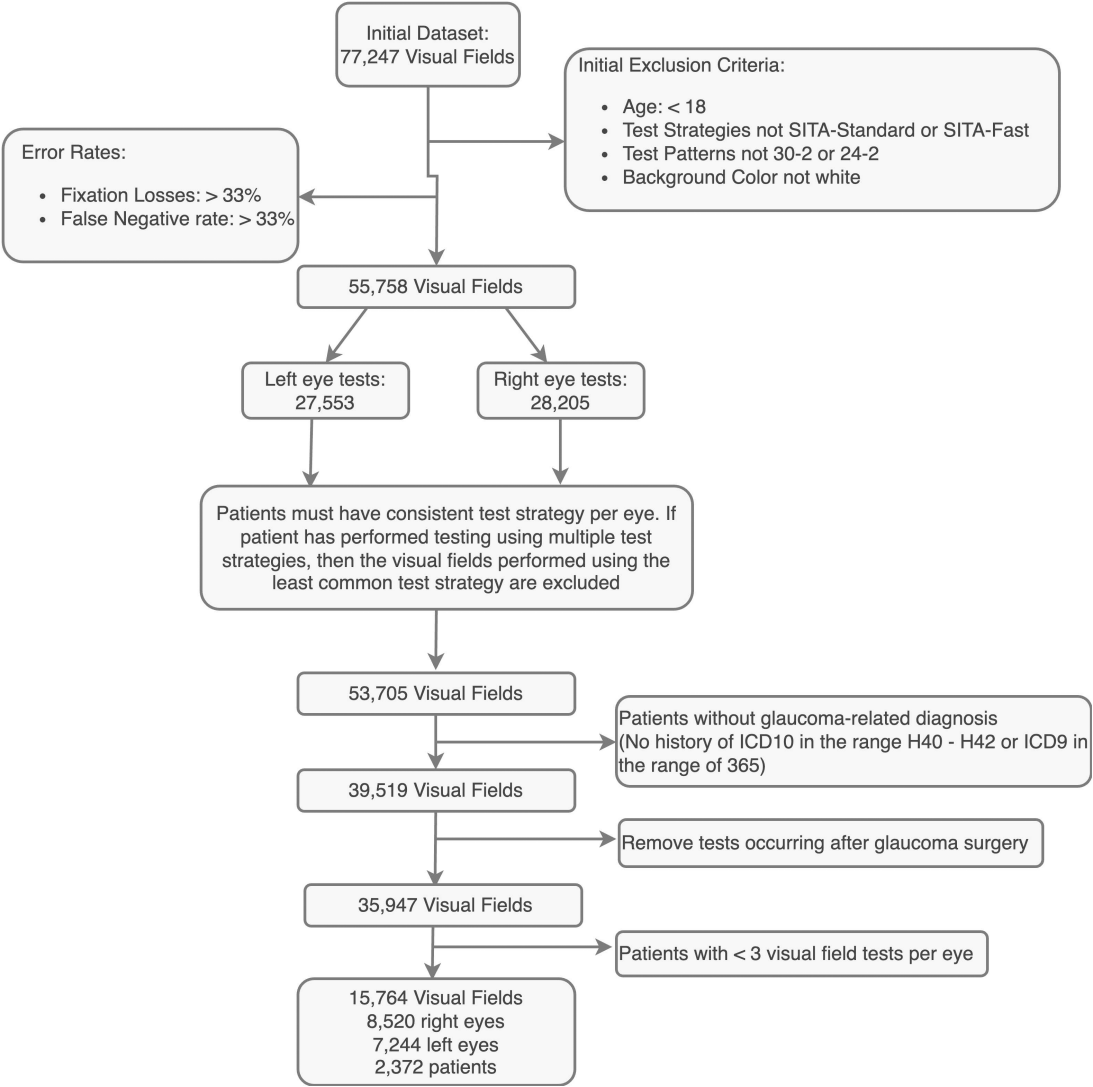
Flow diagram of cohort creation. Detailed cohort creation is depicted in the flow diagram, along with inclusion and exclusion criteria.

Visual field (VF) and electronic health record (EHR) data were retrospectively collected from patients seen at the Byers Eye Institute at Stanford between 2009-2023. **Figure 1** shows the cohort creation process and **Table 1** shows cohort characteristics. All VF tests employed the Humphrey Field Analyzer (Carl Zeiss Meditec, Germany). VF tests administered using a non-white background color, the 10–2 test pattern, or belonging to patients under 18 were excluded. Only reliable tests with fixation and false negative rates below 33% were included, aligning with previous research (11–14). For eyes tested using multiple strategies, (e.g. both SITA-Standard and SITA-Fast tests) the minority test strategy was discarded to ensure all compared tests were consistent. Patients must have a glaucoma-related encounter diagnosis, indicated by an International Classification of Diseases (ICD) code of H40-H42 or ICD9 code beginning with 365. Visual fields conducted on eyes subsequent to glaucoma surgery (Current Procedural Terminology codes in **Supplementary Table 1**) were excluded. Each eye must have at least three visual field tests using the same size and strategy. These criteria resulted in a final set of 15,764 visual fields from 2,372 patients. For evaluation, the cohort was split by patient in a 80:10:10 ratio, resulting in a training set of 12,611, a validation set of 1,577, and a test set of 1,576 visual fields.

**Table 1:** Cohort characteristics.

Characteristic	Slow progressors	Fast progressors	Total
Count (Eye)	3,717	364	4,081
Slope (dB/year)	0.20	-2.51	-0.04
Best recorded visual acuity (logMAR)	0.13 ± 0.33	0.12 ± 0.30	0.13 ± 0.33
Maximum IOP	21.57 ± 7.36	21.58 ± 7.16	21.57 ± 7.34
Cup to Disc Ratio	0.62 ± 0.08	0.62 ± 0.09	0.62 ± 0.08
Central Corneal Thickness	558.95 ± 15.99	558.37 ± 13.93	558.90 ± 15.82
Spherical Equivalent	-2.36 ± 3.24	-2.35 ± 3.51	-2.36 ± 3.27
Age at First Test	60.64 ± 14.86	56.69 ± 16.62	60.29 ± 15.06
Baseline Mean Deviation	-5.03 ± 6.36	-5.96 ± 5.97	-5.11 ± 6.33
	% (N)	% (N)	% (N)
Non-Hispanic White	36.84% (1348)	28.25% (102)	36.07% (1450)
Non-Hispanic Black	2.62% (96)	3.32% (12)	2.69% (108)
Hispanic	8.58% (314)	11.08% (40)	8.81% (354)
Asian	39.63% (1448)	43.77% (158)	40.00% (1606)
Other	10.90% (399)	13.02% (47)	11.09% (446)
Not Given	1.42% (52)	0.55% (2)	1.34% (54)
Gender (Female)	50.74% (1886)	52.47% (191)	50.89% (2077)

### 2.2 Visual field and EHR feature engineering

Visual field data included mean deviation, pattern standard deviation, and pointwise results from the threshold map (dB). The patient’s overall mean deviation slope was determined using subject-level linear regression models, using *SciPy* (V1.13.1) (21) and added as a feature to the dataset. 35 outlier eyes were removed using the Tukey Method (lower bound = Q1 - 1.5•IQR, upper bound = Q3 + 1.5•IQR). Eyes were labeled as fast progressors if mean deviation slope was worse than -1 dB/year.

Features available from EHR included demographic information, clinical eye examination information, ophthalmic and systemic diagnoses and medication usage. Only baseline information from on or before the date of a patient’s first VF test was included. Continuous features included patients’ best recorded visual acuity (VA), intraocular pressure (IOP), refraction spherical equivalent, optic nerve cup-to-disc ratio (CDR), central corneal thickness (CCT), and age at baseline. Demographic features (race/ethnicity, sex) and systemic and ocular medications and diagnoses were encoded as categorical variables. Missing data were handled as follows: missing race was considered “Other”; numerical features from the EHR were imputed with the mean. Simple imputation methods were used as all features had low missingness (<0.6%) except for the spherical equivalent (27%). In total, 431 input features were derived from baseline EHR data. Further feature engineering details are described in Appendix A.1.

### 2.3 Modeling approach

**Figure 2:**
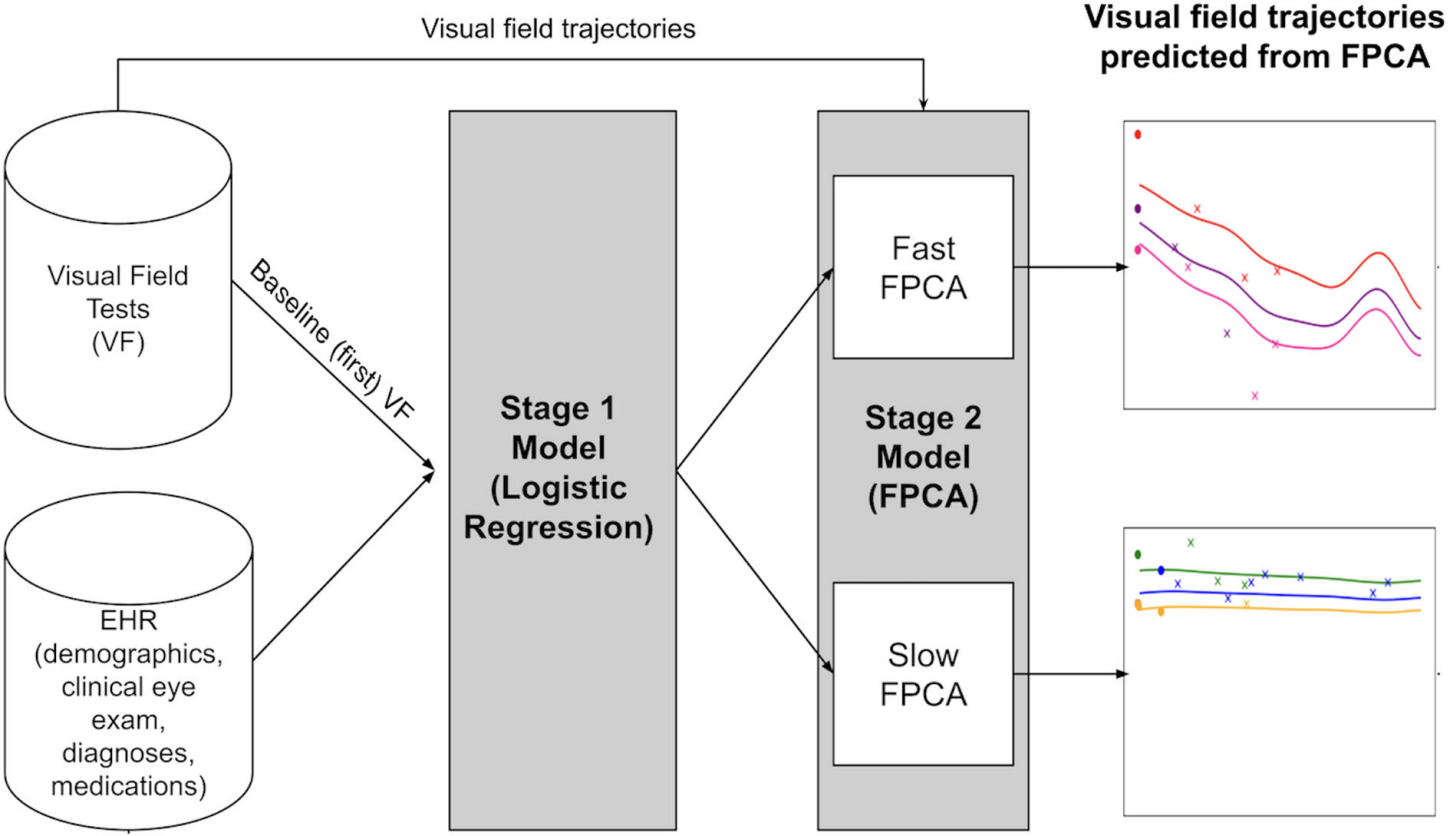
Overview of modeling approach for predicting future visual field using functional principal component analysis and electronic health record information. The proposed two-stage model architecture predicts the Mean Deviation (MD) value at a given timepoint. In Stage 1, a logistic regression model classifies patients into fast and slow progressors using electronic health record (EHR) data and visual fields data from a single visit. Stage 2 involves fitting Functional Principal Component Analysis (FPCA) models using all the Visual Field data. This gives an estimate of the trajectory over the next 10 years, for each progression category.

The modeling approach for predicting the value of future VF MD is summarized in **Figure 2**. We implemented a two-stage model where the output of one stage serves as input for the next, until the final model predicts MD for an individual at any time up to 10 years into the future.

Stage 1: This stage classifies patients into fast/slow progressors using baseline EHR data and first-year VF data (56% of eyes had only one VF as baseline) enabling stratified modeling in subsequent stages. Several candidate models were evaluated (**Table 2**). Logistic regression performed best on the validation set and was chosen as the final Stage 1 model. To address the fast/slow progressor imbalance (8.92% (N = 364) of eyes were fast progressors) the loss function was weighted by the inverse proportion of class frequencies (w = 10.53). Additional experiments with resampling and feature selection techniques were explored to improve performance but ultimately not used in the final model (Appendix A.2, **Supplementary Table 2**). Hyperparameters were tuned using grid search and included the L2 regularization parameter C = 1.

**Table 2:** Classification results for identification of fast progressors.

Candidate Classifier	Accuracy	Balanced accuracy	Recall/ sensitivity	Specificity	Precision (PPV)	NPV	F1 score	AUC	Threshold
Logistic regression	**0.863** **(95% CI: 0.828–0.895)**	**0.696** **(95% CI: 0.601–0.782)**	0.498 (95% CI: 0.310–0.667)	**0.894** **(95% CI: 0.862–0.923)**	**0.285** **(95% CI: 0.167–0.403)**	0.954 (95% CI: 0.932–0.975)	**0.360** **(95% CI: 0.224–0.482)**	**0.773** **(95% CI: 0.687 – 0.845)**	0.65
TabNet	0.528 (95% CI: 0.478–0.578)	0.645 (95% CI: 0.563–0.716)	0.784 (95% CI: 0.625–0.923)	0.506 (95% CI: 0.454–0.558)	0.119 (95% CI: 0.077–0.164)	**0.965** **(95% CI: 0.939–0.989)**	0.206 (95% CI: 0.140–0.275)	0.654 (95% CI: 0.568– 0.730)	0.06
XGBoost	0.754 (95% CI: 0.713–0.797)	0.666 (95% CI: 0.574–0.756)	0.561 (95% CI: 0.375–0.739)	0.770 (95% CI: 0.730–0.811)	0.172 (95% CI: 0.099–0.252)	0.954 (95% CI: 0.930–0.976)	0.262 (95% CI: 0.162–0.366)	0.696 (95% CI: 0.592 – 0.798)	0.03
SVC	0.216 (95% CI: 0.176–0.257)	0.518 (95% CI: 0.452–0.575)	**0.876** **(95% CI: 0.756–0.973)**	0.160 (95% CI: 0.125–0.198)	0.081 (95% CI: 0.052–0.112)	0.938 (95% CI: 0.872–0.986)	0.149 (95% CI: 0.098–0.199)	0.526 (95% CI: 0.428 – 0.632)	0.09

Bold-face numbers denote the best metric across the candidate classifiers.

Stage 2: This stage predicts future MD using VF information, stratified for fast and slow progressors. We employed FPCA, which allows us to decompose irregularly and sparsely collected longitudinal VF data into a mean trajectory, a low-dimensional set of principal modes of variation (functional principal components, FPC), and a set of corresponding subject-specific FPC scores, which express how far an individual’s trajectory deviates from the mean in the direction of a given mode of variation (22). FPCA pools data across subjects over the time domain, harnessing this information to perform individual-level longitudinal predictions at arbitrary future timepoints (Appendix A.3). For fast and slow progressors, we individually predicted future MD trajectories up to 10 years using 1 year of VF exams with the R package *fdapace* (22, 23). Predictions were first discretized into 100 equispaced points along the timeline, then linear interpolation was used to estimate MD between points.

### 2.4 Evaluation

For Stage 1, we calculated the area under the receiver operating characteristic curve (AUROC), area under the precision-recall curve, sensitivity (recall), specificity, positive predictive value (precision), negative predictive value (NPV), and F1 score on the test set. The best model was determined using AUROC, and the classification threshold was chosen to maximize validation set F1 score. Feature importance for the highest-performing model, logistic regression, was determined using the magnitude of the coefficient and the effect size (β/standard error). For final FPCA model directly predicting MD (Stage 2), we calculated Root Mean Squared Error (RMSE) and R^2^ with 95% confidence intervals using non-parametric bootstrapping with 1,000 replicates on the test set, reporting the median values. RMSE approximately measures the average absolute error between predicted and actual MD values, with values closer to 0 indicating higher accuracy. R^2^ quantifies the proportion of variance in the dependent variable explained by the predictors, with values closer to 1 signifying strong predictive power. The FPCA mean functions and first 2 eigenfunctions were examined to provide insight into the FPCA predictions.

## 3 Results

### 3.1 Population characteristics

A total of 15,764 VF tests from 4,081 eyes of 2,372 patients were included. Population characteristics are summarized in **Table 1**. The mean age was 60.29 years and average baseline MD was -5.11 dB. A majority of patients were non-Hispanic White (36.07%) or Asian (40.00%), with approximately equal male and female patients (49.18% and 50.82%, respectively). The mean visual acuity was 0.13 logMAR units, and the mean maximum IOP was 21.57 mmHg. The median number of VF tests per eye was 4. The median time between first and last VF was 1,651 days with an IQR of 1,721 days (Q1 = 953 days, Q3 = 2674 days). 364 eyes were fast progressors (8.92%).

### 3.2 Stage 1: identification of fast progressors using EHR and baseline VF

**Figure 3:**
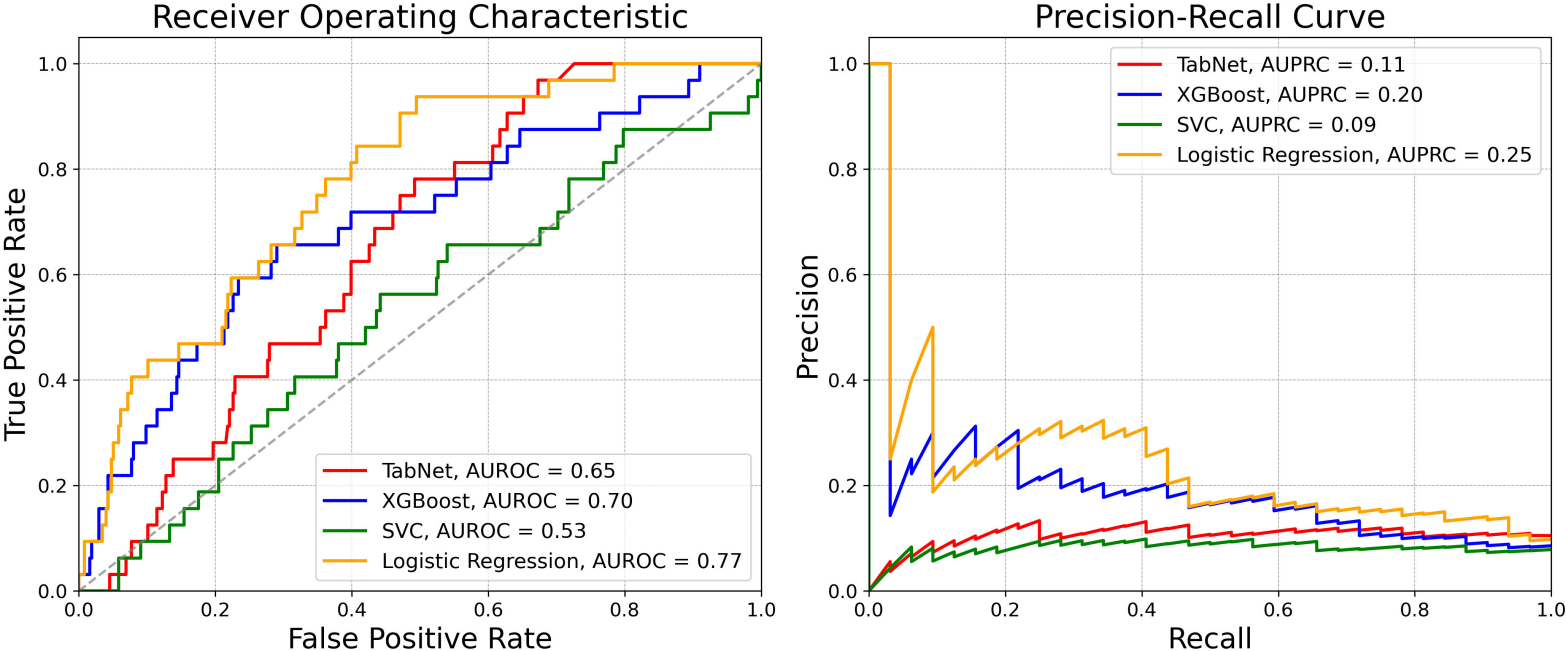
Performance of Stage 1 model to classify fast and slow VF progressors. Receiver operating characteristic curves (left) and precision recall curves (right) across different machine learning models.

Candidate models were trained to classify fast progressors based on baseline VF tests and EHR data, including demographics, clinical examination data (VA, IOP, CCT, spherical equivalent), and ocular and systemic medication usage and diagnoses. Model performance metrics are shown in **Table 2**, and **Figure 3** depicts receiver operating and precision-recall curves. A confusion matrix is shown in **Supplementary Figure 1**. Logistic regression performed best, achieving a 0.77 (95% CI: 0.69–0.85) AUROC for identifying fast progressors. Evaluation of regression coefficients showed the top most important features included age, race, spherical equivalent (refraction), and others (**Supplementary Table 3**).

### 3.3 Stage 2: predicting future mean deviation using FPCA

**Figure 4:**
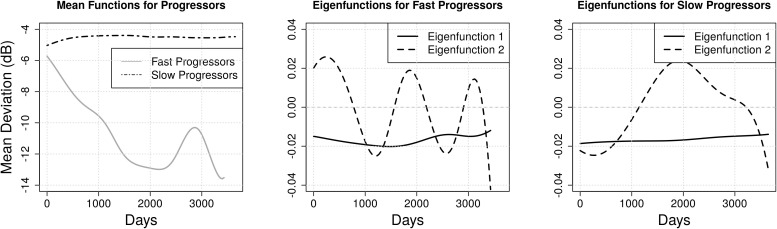
Functional principal component analysis of visual field trajectories. Mean function of both the progression categories (left), first 2 eigenfunctions (middle) which explain 99.5% of the variance for the fast progressors, and first 2 eigenfunctions (right) which explain 99.9% of the variance for the slow progressors. The increased hump in the mean function of the fast progressors is because of a single patient having multiple VF tests around day 3000.

FPCA was conducted using the training MD trajectories for both fast and slow progressors, estimating the population mean MD trajectory and first two eigenfunctions, which represent the dominant modes of variation in VF progression (**Figure 4**). The mean curve for fast progressors exhibits a steep decline, whereas it remains relatively constant for slow progressors. In both categories, the first eigenfunction is flat and represents constant individual-specific variation in MD which does not change over time. However, the second eigenfunction differs across groups; fast progressors with large second FPC scores exhibit higher frequency contrasts, indicating increased trajectory oscillation in comparison to similarly scoring slow progressors, who exhibit more gradual oscillation over the interval.

The fraction of variation explained by each principal component is useful in understanding the relative importance of each mode of variation. For fast progressors, deviations from the mean trajectory in the direction of the first eigenfunction account for 98.5% of variation in observations, while the second eigenfunction explains only an additional 1% of variation. Similarly, for slow progressors, deviations from the mean trajectory in the direction of the first eigenfunction explain 98% of total variation, while the second eigenfunction accounts for 1.9%.

**Figure 5:**
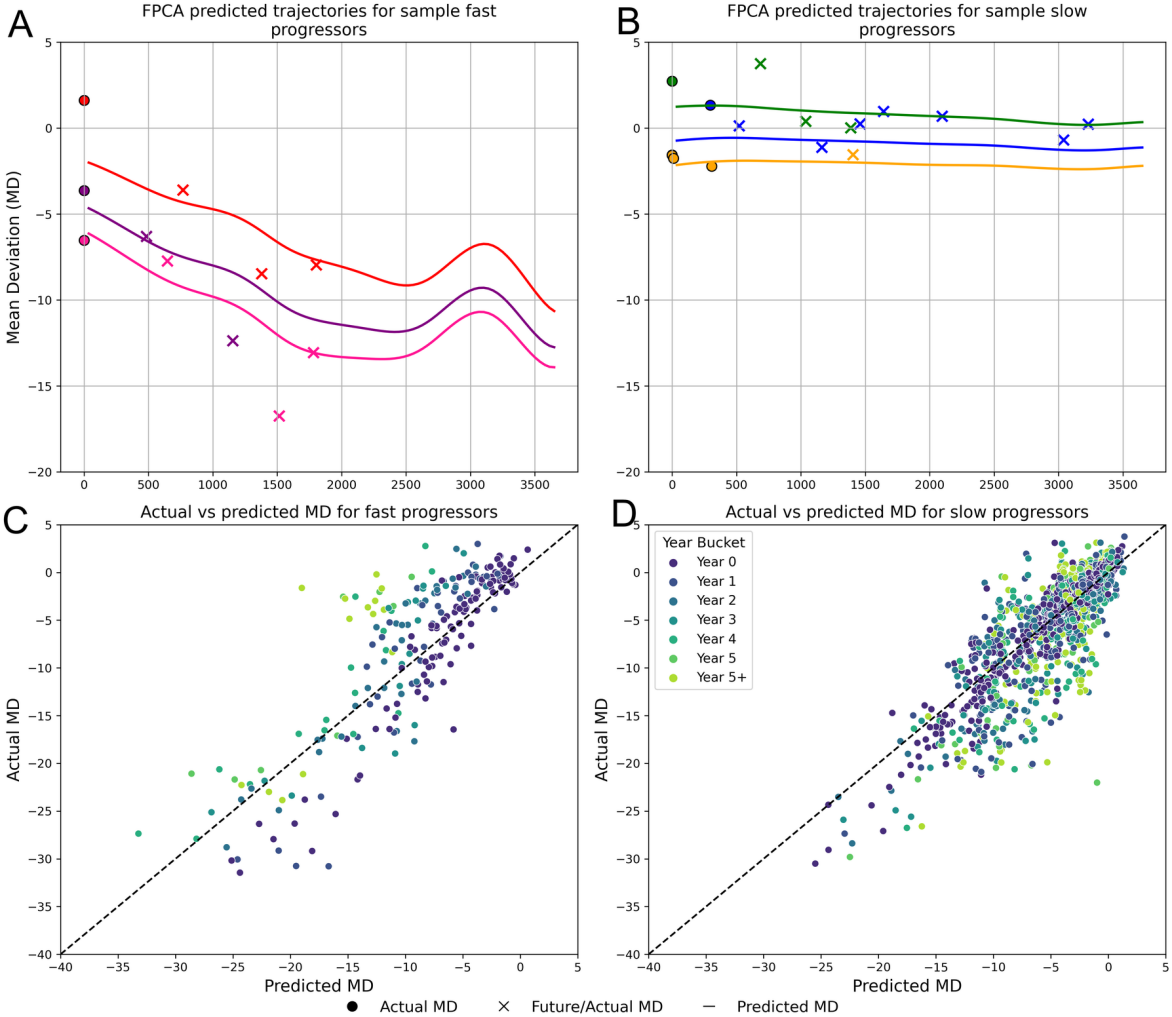
Predicted VF trajectories compared to actual observed VF MD. The figure illustrates example predicted trajectories for Fast progressors **(A)** and Slow progressors **(B)** over a 10-year period. Using one year of test data (indicated by dotted circles), trajectories (indicated by the continuous line) were fitted for each patient employing Functional Principal Component Analysis (FPCA). The crosses represent ground truth future MD values, serving as a comparison to validate the predicted trajectories. **(C, D)** show scatterplots for the actual vs predicted MD values for fast and slow progressors. The points on each plot are distinguished by color, representing different prediction time horizons.

After training the FPCA model, we used the test set to evaluate the model’s ability to predict the next 10 years of MD trajectories, given one year of baseline VF inputs. **Figures 5A, B** provide examples of FPCA trajectory predictions compared with actual future MD values. **Figures 5C, D** display a scatterplot of MD predictions vs actual values for the entire test set of fast and slow progressors, respectively. The FPCA model had an overall RMSE = 3.091, R^2^ = 0.728 for slow progressors and an overall RMSE = 3.665, R^2^ = 0.646 for fast progressors.

Short-term predictions were more accurate than long-term predictions. When the model was evaluated across different future time horizons, predictions over nearer time periods (e.g. 0–1 years, 1–2 years) had higher R² values (**Table 3**). For fast progressors, R² remains robust in the initial years but dips below 0.5 after the fifth year. For slow progressors, R² remains fairly consistent beyond the fifth year.

**Table 3:** RMSE and R^2^ score metrics for the final model’s prediction on the test set for each year-long time interval for future prediction, separated by fast & slow progressor classification.

Time Interval	Fast progressors			Slow progressors			Fast progressors			Slow progressors		
	RMSE	Lower 95% CI	Upper 95% CI	RMSE	Lower 95% CI	Upper 95% CI	R^2^	Lower 95% CI	Upper 95% CI	R^2^	Lower 95% CI	Upper 95% CI
Year 0-1	1.829	1.243	3.051	1.771	1.411	2.043	0.920	0.736	0.960	0.918	0.890	0.950
Year 1-2	3.342	2.243	5.076	3.120	2.569	3.533	0.735	0.398	0.880	0.733	0.630	0.817
Year 2-3	3.771	2.495	5.401	3.274	2.703	3.685	0.654	0.314	0.844	0.716	0.611	0.800
Year 3-4	3.804	2.498	5.364	3.224	2.610	3.678	0.613	0.161	0.847	0.700	0.596	0.795
Year 4-5	4.193	2.224	6.860	3.080	2.315	3.738	0.509	0.089	0.836	0.710	0.608	0.832
Year 5-6	4.452	2.258	7.091	3.118	2.144	3.951	0.515	0.064	0.846	0.717	0.580	0.864
Year >6	4.257	2.858	7.807	3.156	2.333	4.000	0.474	0.080	0.681	0.660	0.525	0.833
Overall (all years)	3.665	1.483	6.552	3.091	1.601	3.813	0.646	0.145	0.946	0.728	0.567	0.933

Overall model performance of the pipeline stratified by race/ethnicity is presented in **Supplementary Table 4**. Across race/ethnicity groups, overall R^2^ varied from 0.708 to 0.774 and overall RMSE varied from 2.655-4.749.

## 4 Discussion

In this study, we present a novel approach to predicting glaucoma patients’ future visual field loss using a multistage architecture which applies functional principal component analysis to visual field data while fusing clinical information from EHR. FPCA allows us to use the entire sample of longitudinal VF tests to better inform individual patients’ future MD trajectories. Using one year of VF data, our model predicts MD over the next 10 years, surpassing prior benchmarks (9, 10). Such models can eventually form the basis of decision support tools that enable clinicians to better personalize glaucoma therapies to prevent irreversible vision loss.

Our work stands out for its innovative use of FPCA to directly predict future VF MD trajectories. In addition, our approach combines clinical information from the EHR with FPCA modeling on VF MD, which constitutes a novel method of fusing data modalities to improve FPCA modeling results. Our method outperforms previous studies which achieved overall RMSE between 4.31 and 4.58 (18, 24). Additionally, our modeling approach addresses key limitations of prior efforts. Our approach only requires one baseline VF for prediction, compared to the several years of baseline data required of many prior models, which can be impractical for clinical use. Moreover, in practice our model allows for dynamic updates with each new assessment, thus accommodating varying amounts of input data without requiring full model retraining. With each new VF test, updated FPCA scores can be determined to produce a new, up-to-date progression prediction.

Our approach to modeling VF progression by combining FPCA with EHR clinical data has broader implications for health informatics beyond ophthalmology. Few prior studies have adopted FPCA for health-related applications (e.g. modeling child growth patterns in underdeveloped countries, assessing the impact of different rehabilitation protocols after stroke, and modeling the progression of Parkinson’s disease) (25–27). Our study adds to this small body of literature and highlights FPCA’s versatility in analyzing longitudinal data, especially in cases when data is sparse, a common challenge in many areas of healthcare. In addition, our approach incorporates the unique strategy of conducting FPCA separately for different patient phenotypes, fast and slow progressors. Implementing stratified FPCA could be valuable to researchers studying heterogeneous populations in other medical fields, allowing for more tailored and accurate modeling of patient subgroups. Finally, our approach of incorporating EHR clinical data into an FPCA pipeline with EHR clinical data provides a framework for assimilating complex types of patient data to yield more accurate predictions.

In our pipeline, FPCA modeling was performed separately for fast and slow progressors; thus, our pipeline incorporated an initial stage which classified patients as fast or slow progressors based on baseline data from the EHR. We prioritized making this classification based on as little baseline data as possible, to maximize the potential applicability of the entire prediction pipeline. The primary purpose of the Stage 1 model was to enhance the FPCA predictions; it was not intended to stand alone in predicting fast vs slow VF progression. Nevertheless, our results (0.77 AUROC, 0.70 balanced accuracy, and 0.36 F1 score) were still somewhat comparable to prior models predicting fast progression requiring far more baseline data, such as in work by Saeedi et al. (11) (0.86 F1 score with logistic regression requiring >= 5 baseline visual field tests), Dixit et al. (15) (0.94 AUC, using a convolutional LSTM requiring >= 4 baseline tests) and Hussain et al. (12) (0.83 AUC and 0.76 F1 score with a deep learning model, requiring 3 baseline tests and OCT imaging. Our Stage 1 model requires only one baseline VF while flexibly using more if available without having to average them: 56% of eyes in our database used only a single VF to make an initial classification, in contrast to the prior studies that leveraged multiple tests and imaging. Minimizing the time needed to collect baseline data for input into models is essential for producing more timely predictions to inform treatment plans before irreversible damage occurs. Although we explored various strategies to improve the Stage 1 model, including SMOTE, LASSO, and others, none significantly enhanced performance. Further methods to improve classification given a small amount of baseline input is a prime area of work for future studies. Additionally, our population had a high degree of imbalance, with a lower proportion of fast progressors (8.92%) than in other studies (12.5% fast progressors (2)), which may have posed additional modeling challenges in our case. Despite this, a crucial advantage of our approach is that since FPCA can naturally incorporate additional VF data as it becomes available, there is the potential for patients to be dynamically reclassified as fast or slow progressors as more data is collected. This flexibility avoids the rigid requirements of methods that rely on a fixed number of baseline inputs (11, 12, 15). Finally, although precision of fast progression prediction remains limited in this work, the threshold of positive prediction can be adjusted according to specific needs and tolerance for false positives in different clinical or research contexts. In most cases, the cost of failing to identify a fast progressor and modeling them as such may be more detrimental than the reverse.

Major challenges in predicting visual field progression are two-fold: predicting over the long horizon and predicting for the minority of patients who are fast progressors. Because fast progressors are a minority of all glaucoma patients, presenting results averaged over all patients risks over-inflating results, which is another reason we have presented results stratified by progression status to maintain maximum flexibility, even where others in prior research have not systematically done so (9). In addition, predicting over long time horizons naturally poses additional challenges, especially for fast progressors whose fields are by definition not stable. Most prior research has focused on predicting over shorter time horizons, such as within 2 years, or grouped predictions together (16, 28). Studies which include longer horizon predictions showed performance for overall populations, which may be skewed towards stable non- and slow-progressors (9). Our results clearly show that modeling likely holds most value for fast progressors only over the first few years. More work is needed for more accurate longer-horizon predictions for fast progressors; however, in clinical practice even apparently stable patients are seen at regular intervals (at least yearly). Thus, in practice, clinicians have more opportunities to update the prognosis with additional data and to intervene if necessary, more frequently than every 3+ years, so the immediate prognosis may be more valuable than a longer-term prognosis. Nevertheless, future studies aimed at predicting visual field progression should always stratify by progression status and by time horizon of the prediction, in order to maximize transparency and evaluation of how models perform under the most challenging circumstances.

Several other limitations remain which provide direction for future research. The cohort was derived from a single center, which may limit generalizability. External validation could help assess our model’s robustness across different populations and clinical settings. Future studies could leverage multicenter registries such as the Sight Outcomes Research Collaborative (SOURCE) to evaluate our approach in an independent population. These larger populations could also enable more granular subgroup-specific FPCA modeling. In the current study, we prioritized FPCA modeling stratified by fast vs slow progression status, but larger sample sizes could also explore FPCA modeling for demographic subgroups as well as formal evaluation of bias and fairness across demographic subpopulations. Ensuring that the model performs well and is equitable across demographics is crucial for clinical acceptance and ethical application. In addition, while our study focused on FPCA as a more flexible modeling alternative to the standard linear-based models for VF trajectories, FPCA assumes smooth deviations from a population mean, which could underfit rare or abrupt patterns in fast progressors. Though FPCA remains a more flexible and interpretable alternative to the clinical standard of linear regression (29, 30), more flexible model architectures could also be explored, such as deep sequence and Bayesian functional models, though deep sequence models reduce interpretability while Bayesian approaches require carefully specified priors. Additionally, our model does not include data from imaging modalities such as fundus photography, nor does it incorporate free-text data from clinical notes, which has been shown to improve predictions (15). These modalities were excluded due to inconsistent availability across patients in our dataset, and to maintain generalizability; however, we acknowledge that their inclusion in future work could enhance model performance and provide richer clinical context upon which to base predictions. Moreover, for future work, FPCA adjusted by covariates (31, 32) can be explored as an alternative to adjusting FPCA for EHR covariates in staged modeling, as well as methods to improve incorporation of time-varying covariates into predictions, as patients’ longitudinal clinical trajectories are complex with many clinical covariates changing over time.

## 5 Conclusion

In conclusion, we have developed a novel method of predicting visual field progression in glaucoma patients using a two-stage model architecture to predict MD trajectories up to ten years in the future. This model fuses clinical data from EHR with baseline VF results and has strong predictive power. Our approach can produce predictions using as little as one visual field but can also incorporate data from multiple VF tests. Our unique longitudinal modeling approach may be applicable for a wide variety of prediction problems across many medical domains. Eventually, models predicting future visual field loss may form the basis of clinical decision support to help physicians personalize therapies for glaucoma patients to avoid vision loss.

## Data Availability

The datasets presented in this article are not readily available because Datasets in this study were derived from electronic health records of patients, which contain protected health information and thus cannot be shared without appropriate data use agreements. Please contact the corresponding author for details. Requests to access the datasets should be directed to Sophia Wang, sywang@stanford.edu.
