# Supplementary material for: Predicting progressive vision loss in glaucoma patients using functional principal component analysis and electronic health records
Source: Front Ophthalmol (Lausanne). 2025 Nov 19;5:1632827. doi: 10.3389/fopht.2025.1632827 (PMC12672302; doi:10.3389/fopht.2025.1632827)
Supplement: Supplementary file 1 [file DataSheet1.pdf]

# Supplementary Methods (Appendix)

## A.1 Additional Feature Engineering Details for Clinical Features from Electronic Health Records

Visual Acuity (VA) was transformed to standard logarithm of the minimum angle of resolution (logMAR) units and included measurements with and without correction, and with auto- or manifest refraction. The minimum (best recorded) VA value across these types of measurements was taken for each patient's eye examination per unique day. Visual acuities across multiple encounter days were summarized into the patient's best, worst, median and most recent VA. IOP measurements were determined from each eye examination, with outliers exceeding 100mmHg removed. IOP values across multiple encounters were summarized into best, worst, median and most recent. Spherical equivalent was determined from documented glasses prescription or auto- or manifest refraction. For patients with multiple refractions, the minimum spherical equivalent was taken. The most recent CCT values were identified, with outliers < 300 considered missing. CDR was summarized into best, worst, median, and most recent. Age at the time of baseline VF was determined. Continuous features were standardized. Mean imputation was performed for missing values. Categorical features with <1% variance were eliminated.

## A.2 Stage 1 Logistic Regression Additional Resampling and Feature Selection Techniques

We explored several techniques to improve model performance given our high dimensional dataset of over 400 features. Synthetic minority oversampling technique (SMOTE) was used to address class imbalance by synthetically oversampling the minority class, helping the model learn a more balanced decision boundary. LASSO (L1 regularization) was applied to perform automatic feature selection by shrinking irrelevant feature coefficients to zero, which can help reduce overfitting and improve interpretability in high-dimensional settings. Recursive Feature Elimination (RFE) was used to iteratively remove less important features based on model performance, allowing us to identify a smaller subset of the most informative predictors. We also implemented undercomplete autoencoders to learn a compressed, denoised representation of the data that could reduce noise and redundancy before classification. Ultimately, none of these techniques significantly improved our final model and hence were not used.

## A.3 Functional Principal Components Analysis

In the training population, Functional Principal Components Analysis (FPCA) was used to estimate the population mean and covariance functions of MD trajectories, with separate models for slow and fast progressors. The Principal Analysis by Conditional Expectation (PACE) algorithm conducts eigenanalysis of the covariance surface and yields the primary modes of variation, known as eigenfunctions [19]. Projecting a given individual's trajectory data onto the k-th eigenfunction results in the corresponding k-th principal component score for that individual. For our models, we truncate the eigenexpansion at $K=2$ functional principal components, as this explains over 99.5% (99.9%) of the variation in the original fast (slow) progressors' data.

For any patient for which we wish to predict the MD trajectory, principal component scores are first estimated from the input visual fields through conditional expectation. Then, each patient's MD trajectory is estimated by adding a linear combination of the product of the principal component scores and their respective eigenfunctions to the estimated population mean trajectory. This process enables the prediction of each patient's MD trajectory over the specified future time period.

# Supplemental Tables

**Supplemental Table 1.** Surgery codes used for VF exclusion

| CPT Codes |
|---|
| 65820, 65850, 65855, 65860, 66150, 66155, 66160, 66165, 60000, 69999, 66170, 66172, 66174, 66175, 66179, 66180, 66183, 66184, 66185, 66220, 60000, 69999, 66250, 66500, 66505, 66600, 66605, 66625, 66630, 66635, 66680, 66682, 66700, 66710, 66711, 66720, 66740, 66761, 66762, 66770, 66825, 66830, 66840, 66850, 66852, 66920, 66930, 66940, 66982, 66983, 66984, 66985, 66986, 66987, 66988, 66989, 66991, 67250, 67255, 0191T, 0376T, 0474T, 0253T, 0449T, 0450T, 0192T |

**Supplemental Table 2**. Results of Stage 1 logistic regression additional resampling and feature selection techniques

| | **Accuracy** | **Balanced Accuracy** | **Recall / sensitivity** | **Specificity** | **Precision (PPV)** | **NPV** | **F1 score** | **AUC** |
|---|---|---|---|---|---|---|---|---|
| LR with SMOTE | 0.824 | 0.575 | 0.281 | 0.870 | 0.155 | 0.934 | 0.200 | 0.575 |
| LR with LASSO | 0.814 | 0.570 | 0.281 | 0.859 | 0.145 | 0.934 | 0.191 | 0.570 |
| LR with RFE | 0.238 | 0.544 | 0.906 | 0.181 | 0.086 | 0.958 | 0.157 | 0.544 |
| LR with undercomplete autoencoders | 0.654 | 0.512 | 0.344 | 0.681 | 0.084 | 0.924 | 0.135 | 0.512 |

LR: logistic regression. SMOTE: synthetic minority oversampling technique. LASSO: Least absolute shrinkage and selection operator. RFE: recursive feature elimination.

**Supplemental Table 3**. Top most important features for Stage 1 (fast progression) classification

| Rank | Stage 1 LR | Coefficient | Effect Size (β/SE) |
|---|---|---|---|
| 1 | Glaucoma suspect, bilateral | 0.664 | 0.158 |
| 2 | HYDROCODONE-ACETAMINOPHEN 5-325 MG PO TABS | 0.414 | 0.101 |
| 3 | Spherical equivalent (refraction) | 0.393 | 0.143 |
| 4 | Race White | 0.336 | 0.082 |
| 5 | Age (standardized) | 0.315 | 0.077 |
| 6 | LATANOPROST 0.005 % OPHT DROP | -0.314 | -0.078 |
| 7 | XALATAN 0.005 % OPHT DROP | -0.310 | -0.077 |
| 8 | Sex Male | 0.309 | 0.076 |
| 9 | ASPIRIN 81 MG PO TBEC | 0.300 | 0.074 |
| 10 | VITAMIN C PO | 0.291 | 0.072 |

**Supplemental Table 4: Overall visual field prediction performance stratified by race/ethnicity**

| | Overall $R^2$ | Fast progression $R^2$ | Slow Progression $R^2$ | Overall RMSE | Fast RMSE | Slow RMSE | Fast progression N eyes | Slow progression N eyes |
|---|---|---|---|---|---|---|---|---|
| **Non-Hispanic White** | 0.742 | 0.698 | 0.746 | 2.743 | 4.311 | 2.578 | 13 | 142 |
| **Non-Hispanic Black** | 0.398 | na | 0.406 | 4.749 | 3.201 | 4.831 | 1 | 9 |
| **Non-Hispanic Asian** | 0.708 | 0.656 | 0.704 | 3.478 | 5.024 | 3.165 | 27 | 139 |
| **Hispanic (of any race)** | 0.765 | 0.695 | 0.805 | 2.655 | 4.387 | 1.852 | 8 | 21 |
| **Other** | 0.774 | 0.744 | 0.744 | 2.811 | 5.248 | 2.377 | 7 | 41 |

# Supplemental Figures

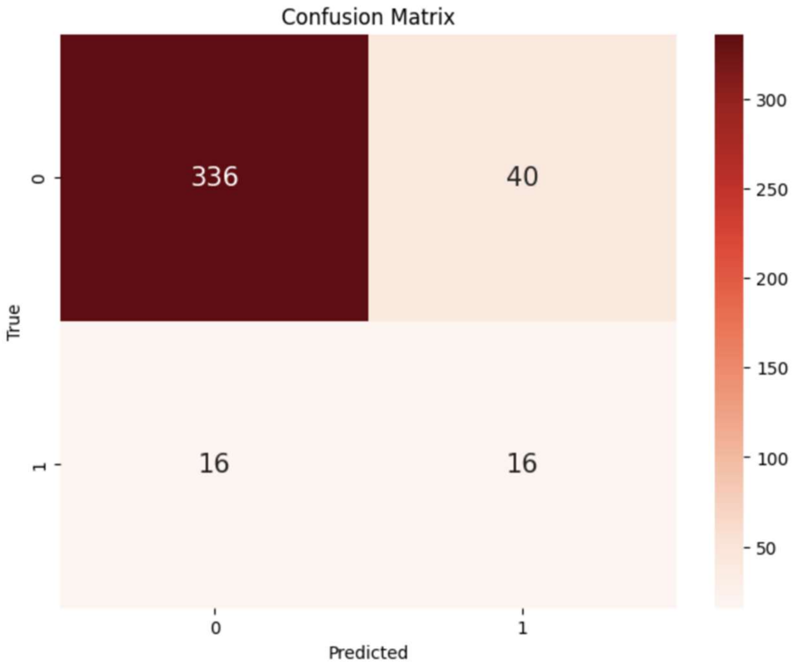


**Supplemental Figure 1. Confusion matrix for Stage 1 model, classification of fast progressors.** The figure depicts the confusion matrix on the test set for the Stage 1 model (logistic regression) which predicts fast progressors (1) vs slow progressors (0) on visual field based on baseline EHR features.
